# A Yield Stress and Work Hardening Model of Al-Mg-Si Alloy Considering the Strengthening Effect of β” and β’ Precipitates

**DOI:** 10.3390/ma16227183

**Published:** 2023-11-16

**Authors:** Xiaoyu Zheng, Qi Huang, Hong Mao, Kai Li, Namin Xiao, Xingwu Li, Yong Du, Yuling Liu, Yi Kong

**Affiliations:** 1 State Key Laboratory of Powder Metallurgy, Central South University, Changsha 410083, China; xiaoyu_zheng@csu.edu.cn (X.Z.); qihuang0908@163.com (Q.H.); leking@csu.edu.cn (K.L.); yong-du@csu.edu.cn (Y.D.); 2 College of Mechanical Engineering, Hunan Institute of Science and Technology, Yueyang 414006, China; paomajun@163.com; 3 Beijing Institute of Aeronautical Materials, Aero Engine Corporation of China, Beijing 100095, China; nmxiao@outlook.com (N.X.); lxwdjh@163.com (X.L.)

**Keywords:** aluminum alloys, precipitates, homogenization, crystal plasticity finite element method, crystal orientation

## Abstract

Precipitates are the primary source of strength for the Al-Mg-Si alloy. Aluminum alloy in the peak-aged state mainly contains β” and β’ precipitates. Most of the literature has only considered the strengthening effect of β”. Here, we develop a single-crystal intensity model including both precipitate enhancement effects for the first time. This model was subsequently implemented into a crystal plastic finite-element method to model the uniaxial tensile process of a polycrystalline aggregate model of Al-Mg-Si alloy. The simulation results for uniaxial stretching are in good agreement with the experimental results, confirming that the constitutive parameters used for the single-crystal strength model with two precipitates are based on realistic physical implications. Furthermore, by comparing the uniaxial tensile simulation results of a peak-aged alloy considering the actual precipitated phase composition of the alloy with those assuming that the precipitated phase is only the β” phase, the predicted tensile strength of the former is around 5.65% lower than that of the latter, suggesting that the two kinds of precipitation should be separately considered when simulating the mechanical response of Al-Mg-Si alloy. It is highly expected that the present simulation strategy is not limited to Al-Mg-Si alloys, and it can be equally applied to the other age-enhanced alloys.

## 1. Introduction

Aged aluminum alloys have been widely used in aerospace, automotive, shipbuilding, and other fields because of their high strength and light weight [1,2,3,4]. The research on their strengthening mechanism and properties has attracted much attention [5,6,7,8,9]. The high strength of the aged aluminum alloy is mainly due to the second-phase particles, which precipitate in the supersaturated solid solution matrix and act as an obstacle to the dislocation motion [10,11,12,13].

The Al-Mg-Si (6XXX-series) aluminum alloys are a class of precipitation hardening materials containing high concentrations of magnesium and silicon solutes. The typical sequence of precipitation for Al-Mg-Si alloys is SSSS (super-saturated solid solution) → Atomic clusters → GP zones → β” → β’ → β [14]. Previous studies have shown that the elongated needlelike coherent β”-precipitates and rod-shaped semi-coherent β’-precipitates in the ${\langle 001\rangle }_{\mathrm{Al}}$ directions are considered to be the main sources of hardening [15]. Usually, these two precipitates are uniformly distributed on ${\left\{111\right\}}_{\mathrm{Al}}$. It is generally believed that β” has a more significant strengthening effect than β’ [16,17,18]. Previous work [15] has quantitatively analyzed the strengthening effects of β’ and β” by means of experimental characterization combined with the macro strength model of AA6XXX-series aging aluminum alloy. The samples of Al-0.66Mg-0.41Si (wt.%) alloy were cast, homogenized, rolled, solution-heat-treated, quenched to room temperature with water, and immediately aged at 180 °C for 3 h and 6 h, respectively, to obtain under-aged and peak-aged alloys.

In the past 20 years, based on the interaction mechanism between the precipitate and dislocation, a lot of scholars have established many advanced yield strength models of aging-strengthened aluminum alloys on the premise of simplifying the type and shape of precipitates. According to the dislocation theory, Myhr et al. [19] evaluated the yield strength and hardness of Al–Mg–Si alloys from the hindrance of solute atoms and precipitates to dislocation movement. Esmaeili et al. [20] further divided the yield strength model into a strong obstacle model and a weak obstacle model according to the characteristics of the interaction between precipitates and dislocations, and gave the applicability of the two models. Holmedal [21] also considered the statistical particle-size and shape distributions of the precipitates to build a precipitation strengthening model, which solved the problem of low-strength calculation due to the assumption that the morphology of the precipitates was spherical. These yield strength models can then be combined with crystal plasticity to simulate the tensile or shear response of the aluminum alloys.

The crystal plasticity method is a systematic meso-mechanical method, which connects the microstructure and properties of materials. The main purpose of the crystal plastic constitutive theory is to establish a plastic flow model, a work hardening model, and an evolution model of internal variables (such as slip system strength and dislocation density) conforming to the plastic deformation mechanism. The pioneering work of the crystal plasticity method was performed by Taylor [22] for face-centered cubic (FCC) polycrystals subject to large plastic strains, and its complete framework was established by Rice, Hill, and Mandel et al. [23,24,25]. Later, Peirce, Asaro, and Kalidindi et al. [26,27,28] further improved the crystal plasticity model and used the finite element method for numerical simulation, which marks the birth of the crystal plasticity finite element method (CPFEM). With the development of computer technology, Harren and Becker et al. [29,30] simulated the mechanical properties of polycrystalline materials by means of crystal plasticity. At present, thanks to the proposal of many open-source programs [31,32,33], crystal plasticity is widely used and has become a mature numerical simulation method [34,35]. 

However, so far, the constitutive equations used in crystal plasticity simulations have only considered contributions from individual precipitates, but the types of precipitates have not been carefully distinguished. Therefore, the aim of the current work is mainly to build a single-crystal strength model including the strengthening effects of two precipitates based on the experimental data, and to simulate the plastic deformation of the material by using the crystal plastic finite element method. Firstly, the uniaxial tensile experimental test results used to verify the simulation results of the subsequent analysis are presented. Then, the strength model and the constitutive relation of crystal plasticity are constructed. Finally, the above model is numerically realized through finite element simulation, and the calculations with various orientation characteristics are set for comparative analysis to explore the error caused by the assumption of a single precipitate.

## 2. Material Characterization

The materials used in this work come from previous work [15]. Firstly, the ingots with a chemical composition of Al-0.66Mg-0.41Si-0.11Fe (wt.%) were homogenized at 500 °C for 12 h, and hot-rolled and cold-rolled to obtain 1 mm thick sheets. Then, after solid solution heat treatment at 550 °C, the water was quenched to room temperature. Finally, under-aged alloys and peak-aged alloys were obtained by aging at 180 °C for 3 and 6 h. Through a series of characterizations of under-aged and peak-aged alloys with different aging times only, the composition, microstructure, and nanostructure of the materials were examined. Under-aged alloy contains only β” precipitates, and peak-aged alloy contains β” and β’ precipitates. The information obtained from the experiment is used in the subsequent finite element simulation to determine part of the material parameters and for the construction of geometric models, mainly including the average diameter (*d*) of crystal grains, matrix composition, the volume fraction (*f*) of precipitates, and the average section radius ($r_{p}$) on ${\left\{111\right\}}_{\mathrm{Al}}$ of precipitates, as shown in Table 1.

**Figure 1:**
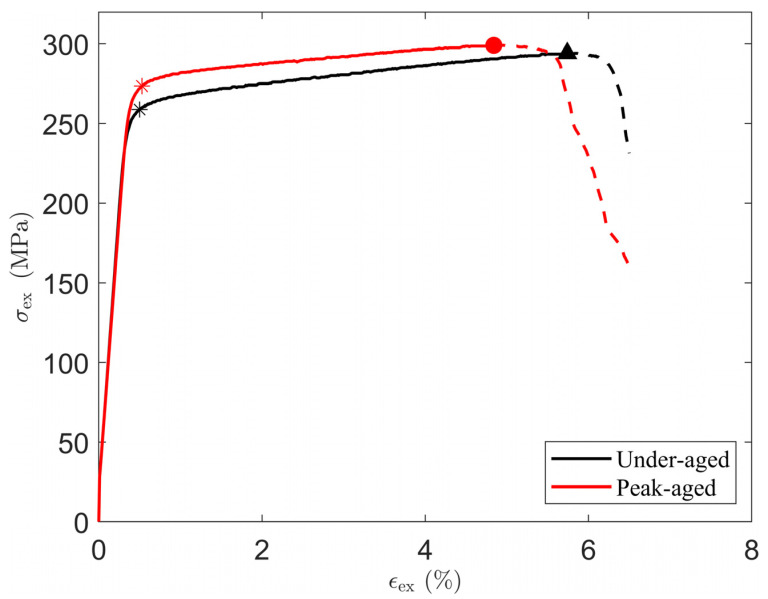
True stress–strain curves of under-aged and peak-aged alloys in uniaxial tensile experimental tests, converted from engineering stress–strain curves in [15]. The data after the maximum tensile strength are represented by a dotted line, which means that the material was damaged and it failed at this stage. The symbol * is used to indicate the yield strength, and the dot and triangle are used to indicate the maximum tensile strength of the two materials, respectively.

The uniaxial tensile test is the most common method to evaluate the mechanical properties of aging-strengthened aluminum alloys in the laboratory. The complete stress–strain curve (true stress–strain curve) of the under-aged alloy and the peak-aged alloy obtained through the test is shown in Figure 1. Reference [15] documents the test standards followed for tensile testing. Tensile tests were performed using an Instron 3369 testing machine (Instron, Kawasaki, Japan) at room temperature at a constant crosshead speed of 5 mm/min. A tensile specimen with a gauge length of 25.0 mm and a width of 6.0 mm was cut from a 1.0 mm thick rolled plate, with its long axis parallel to the rolling direction. Three uniaxial tensile tests were then performed on specimens in both aging states. The results were averaged after excluding specimens that prematurely failed due to casting defects [15].

The effective area of the true stress–strain curve prior to material failure is shown as a solid line in the figure. The Young’s modulus E, 0.2% offset yield strength $\sigma_{y}$, yield strain $\epsilon_{y}$, and tensile strength $\sigma_{\max}$ with corresponding strain $\epsilon_{\max}$ can be obtained from the stress–strain curve, which are recorded in Table 2. In addition, work hardening occurs after yielding and continues until the stress reaches $\sigma_{\max}$. The maximum work hardening $\sigma_{w,\max}$ is the difference between $\sigma_{\max}$ and $\sigma_{y}$. The alloy used in this work is similar to AA 6063-T832 [36] in terms of the magnesium and silicon content, heat treatment process, and yield stress and tensile strength in the peak-aged state.

In addition, considering that the material has not undergone excessive deformation processing from the casting of the alloy until the preparation of the uniaxially stretched sample, and the experimental data are the average of the simulation results of several different samples, it can be assumed that the material orientation is random.

## 3. Modeling

The numerical method used in this paper is the CPFEM to simulate the uniaxial tensile deformation of the polycrystalline aggregate. The finite element (FE) simulation is mainly carried out using the commercial finite element software ABAQUS (2022). The theoretical part mainly includes the strength model and crystal plasticity constitutive model. The strength model is mainly based on the work of Esmaeili et al. [20], and a model considering the strengthening effect of two kinds of precipitates is constructed. Subsequently, the strategy of Khadyko et al. [37] is used to convert the macroscopic yield stress into the initial flow stress of the single crystal. The crystal plastic model is mainly used to describe the strain hardening behavior of materials, to capture the possible locations of crack initiation, and to explore the effects of microstructure parameters on mechanical properties.

### 3.1. Strength Model

The yield strength of the Al-Mg-Si Alloys $\sigma_{y}$ is assumed to be obtained through the linear superposition of several mechanisms [19,20,38]:

(1) $$\sigma_{y}=\sigma_{0}+\sigma_{\mathrm{ss}}+\sigma_{\mathrm{ppt}},$$

where $\sigma_{0}$ is the initial yield strength of polycrystalline pure aluminum, $\sigma_{\mathrm{ss}}$ is the contribution from alloying elements in solid solution, and $\sigma_{\mathrm{ppt}}$ is the strengthening contribution from precipitation hardening.

Generally, $\sigma_{0}$ is negatively related to the grain size, which satisfies the Hall–Petch relationship:

(2) $$\sigma_{0}=\sigma_{i}+k_{y}d^{-1/2},$$

where $\sigma_{i}$ is the intrinsic strength of Al, $k_{y}$ is the Hall–Petch constant, and d is the diameter of the grain.

**Figure 2:**
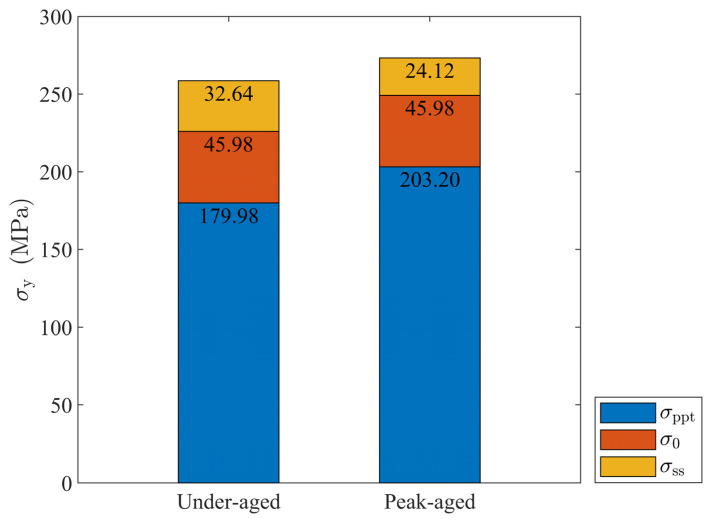
Individual terms for yield strength of under-aged and peak-aged Al-Mg-Si alloys.

The solid solution strengthening is provided by the solid solution elements in the matrix, and the total strengthening effect is superimposed by the contribution of each element. $\sigma_{\mathrm{ss}}$ can be expressed as [19]

(3) $$\sigma_{\mathrm{ss}}=\underset{i=\mathrm{Mg},\mathrm{Si}}{\sum }k_{i}C_{i}^{2/3},$$

where $C_{i}$ is the concentration of a specific alloying element in the solid solution and $k_{i}$ is the corresponding scaling factor. Here, the solid solution strengthening contribution of those trace elements is not considered. It is worth noting that the yield strength calculated from Formula (1) or through a uniaxial tensile test is based on the assumption that the material is macroscopically isotropic. Table 3 lists all the parameter values used in this section. The experimental data in Table 1 and Table 2 are used in Formulas (1)–(3) to obtain $\sigma_{0}$, $\sigma_{\mathrm{ss}}$, and $\sigma_{\mathrm{ppt}}$, and the results are shown in Figure 2. Later, $\sigma_{\mathrm{ppt}}$ is used to obtain the hindrance of precipitates to lattice slip on the single-crystal level.

It can be seen from Figure 2 that the age strengthening contribution of the two alloys reaches about 70%, and the solid solution strengthening effect decreases as Mg and Si enter the precipitates and the other second phase such as AlFeSi [15]. In addition, the contribution of the solid solution strengthening effect is only about 10%, and it can be considered that the solid solution basically does not strengthen the alloy in this work.

In reality, the main mechanism of plastic deformation of aluminum alloy is the resolved shear stress on the slip system exceeding the initial slip resistance $\tau_{y}$ required for activation of the slip system. $\tau_{y}$ is the most critical physical quantity that reflects the strength of a material in the theory of crystal plasticity. The expression of $\tau_{y}$ is similar to $\sigma_{y}$:

(4) $$\tau_{y}=\tau_{0}+\tau_{\mathrm{ss}}+\tau_{\mathrm{ppt}},$$

where the contributions to the critical resolved shear stress from the intrinsic strength of aluminum, the solid solution, and the particles are denoted as $\tau_{0}$, $\tau_{\mathrm{ss}}$, and $\tau_{\mathrm{ppt}}$, respectively. $\tau_{0}$ is a constant derived from the literature. $\tau_{\mathrm{ss}}$ can be expressed as [39]

(5) $$\tau_{\mathrm{ss}}=\underset{i=\mathrm{Mg},\mathrm{Si}}{\sum }k_{i}^{\prime }C_{i}^{2/3},$$

where $k_{i}^{\prime }$ and $k_{i}$ have basically the same meaning but correspond to different material scales. It is assumed that there is a linear relationship between $k_{i}^{\prime }$ and $k_{i}$, $k_{i}=M_{s}k_{i}^{\prime }$. $M_{s}$ is the coefficient to be calibrated, which depends on the distribution position of solid solution elements and the microstructure characteristics of the material.

The work of Khadyko et al. [37] showed that $\tau_{\mathrm{ppt}}$ and $\sigma_{\mathrm{ppt}}$ were connected through Taylor factor *M*:

(6) $$\tau_{\mathrm{ppt}}=\sigma_{\mathrm{ppt}}/M$$

Therefore, Formula (4) is finally expressed as

(7) $$\tau_{y}=\tau_{0}+\sigma_{\mathrm{ss}}/M_{s}+\sigma_{\mathrm{ppt}}/M$$

It is worth noting that *M* is a value strongly related to the statistical characteristics of the crystallographic orientation. Khadyko et al. [37] give the possible range of values in aluminum alloys. This parameter value is calibrated in the finite element realization of the crystal plastic constitutive process below. It is reasonable to assume that under-aged and peak-aged alloys have the same *M* because the grain does not undergo rotation and large deformation during aging. A general value range of *M* (2.4~3.1) was given by Bahrami et al. [40].

**Table 3:** Parameters of strength model.

Parameter	Value	Ref.
$\sigma_{i}\left(\mathrm{MPa}\right)$	10.0	[41]
$\tau_{0}\left(\mathrm{MPa}\right)$	17.0	[9]
$k_{y}\left(\mathrm{MPa}\cdot m^{1/2}\right)$	0.326	[42]
$k_{\mathrm{Mg}},k_{\mathrm{Si}}\left(\mathrm{MPa}/\mathrm{wt}.{\%}^{2/3}\right)$	29.0, 66.3	[19]

### 3.2. Crystal Plasticity Constitutive Model

The crystal structure of the Al phase in Al-Mg-Si alloy is FCC, which contains only one set of slip systems $\left\{111\right\}\langle 110\rangle$, including 12 slip systems involved in plastic deformation. The list of all the available slip systems for FCC can be referred to in Bassani [43]. When the stress on the slip system reaches the critical value required for slip, plastic deformation occurs.

The theory of crystal plasticity has two main aspects. One is the description of the plastic deformation behavior of a single-crystal grain, and the other is the obtaining of the macroscopic plastic response in the category of polycrystals by coordinating the deformation between individual crystal grains.

#### 3.2.1. Single-Crystal Constitutive Equations

The model used in this work was originally developed by Asaro [44]. Under the action of external load, the crystal grains of polycrystalline materials will deform and rotate. The deformation gradient F of each individual crystal can be decomposed into a lattice deformation gradient $F^{*}$ and a plastic deformation gradient $F^{p}$:

(8) $$F=F^{*}F^{p},$$

where $F^{*}$ reflects the elastic deformation and rotation of the crystal lattice, and $F^{p}$ denotes the plastic deformation of the crystal lattice along the slip direction.

The velocity gradient l is defined as

(9) $$\begin{array}{c} l=\dot{F}\cdot F^{-1}={\dot{F}}^{*}\cdot F^{*-1}+F^{*}\cdot {\dot{F}}^{p}\cdot F^{p-1}\cdot F^{*-1}\\ \left\{\begin{array}{c} l^{*}={\dot{F}}^{*}\cdot F^{*-1}\\ L^{p}={\dot{F}}^{p}\cdot F^{p-1}\\ l^{p}=F^{*}\cdot {\dot{F}}^{p}\cdot F^{p-1}\cdot F^{*-1} \end{array}\right. \end{array},$$

where $l^{*}$ and $l^{p}$ are the elastic velocity gradient and plastic velocity gradient defined in the current configuration, respectively, and $L^{p}$ is the plastic velocity gradient defined in the intermediate configuration.

For the plastic deformation due to slip, the relationship between the plastic velocity gradient and the plastic slip rate ${\dot{\gamma }}^{\alpha }$ on each slip system α satisfies the following relationship:

(10) $$\left\{\begin{array}{c} L^{p}=\underset{\alpha }{\sum }{\dot{\gamma }}^{\alpha }s^{\alpha }⊗m^{\alpha }\\ l^{p}=\underset{\alpha }{\sum }{\dot{\gamma }}^{\alpha }s^{*\alpha }⊗m^{*\alpha } \end{array}\right.,$$

where the unit vectors $s^{\alpha }$ and $s^{*\alpha }$ are the slip directions in the reference configuration and the deformation configuration, respectively, and the unit vectors $m^{\alpha }$ and $m^{*\alpha }$ represent the normal of the slip surface in these two configurations, respectively. $s^{*\alpha }$ and $m^{*\alpha }$ can be easily obtained by $s^{\alpha }$ and $m^{\alpha }$, respectively:

(11) $$\left\{\begin{array}{c} s^{*\alpha }=F^{*}\cdot s^{\alpha }\\ m^{*\alpha }=m^{\alpha }F^{*-1} \end{array}\right.$$

${\dot{\gamma }}^{\alpha }$ is approximated by a power law, assuming that plastic flow occurs under all non-zero stresses without any yield condition or loading/unloading condition:

(12) $${\dot{\gamma }}^{\alpha }={\dot{\gamma }}_{0}{\left(\frac{\left|\tau^{\alpha }\right|}{g^{\alpha }}\right)}^{\frac{1}{m}}\mathrm{sgn}\left(\tau^{\alpha }\right),$$

where ${\dot{\gamma }}_{0}$ is the reference shear strain rate, $g^{\alpha }$ is the critical resolved shear stress, and m is the slip rate sensitivity parameter. It is assumed that the $g^{\alpha }$ of all slip systems has the same initial value $\tau_{y}$. According to Schmid’s law, the resolved shear stress $\tau^{\alpha }$ on the *α*th slip system can be defined as

(13) $$\tau^{\alpha }=\left(m^{*\alpha }⊗s^{*\alpha }\right):\tau =m^{*\alpha }\cdot \tau \cdot s^{*\alpha },$$

where $\tau =\det\left(F\right)\sigma \approx \sigma$ is the Kirchhoff stress tensor. By introducing $P^{\alpha }=\mathrm{sym}\left(s^{*\alpha }⊗m^{*\alpha }\right)$, $Q^{\alpha }=\mathrm{skew}\left(s^{*\alpha }⊗m^{*\alpha }\right)$, and $D=\mathrm{sym}\left(l\right)$, ${\dot{\tau }}^{\alpha }$ can be expressed as

(14) $${\dot{\tau }}^{\alpha }=\left(L:P^{\alpha }+Q^{\alpha }\sigma +\sigma {\left(Q^{\alpha }\right)}^{T}\right):\left(D-\underset{\beta }{\sum }{\dot{\gamma }}^{\beta }P^{\beta }\right),$$

where L is the fourth-order elastic modulus tensor, and for FCC materials, it only contains three independent constants $C_{11}$, $C_{12}$, and $C_{44}$.

The strain hardening equation is

(15) $${\dot{g}}^{\alpha }=\overset{12}{\underset{\beta =1}{\sum }}h_{\alpha \beta }\left|{\dot{\gamma }}^{\beta }\right|,$$

where $h_{\alpha \beta }$ are the slip hardening moduli, and the sum ranges over all activated slip systems. For FCC materials, $\left[h_{\alpha \beta }\right]$ is formally a 12 × 12 matrix, the components on the main diagonal indicate self-hardening moduli, and the components on the non-main diagonal indicate latent hardening moduli. A simple form of $h_{\alpha \beta }$ is

(16) $$h_{\alpha \beta }=h\left(\alpha ,\alpha \right)\left[q+\left(1-q\right)\delta_{\alpha \beta }\right]=\left\{\begin{array}{cc} h\left(\alpha ,\alpha \right) & \beta =\alpha \\ qh\left(\alpha ,\alpha \right) & \beta \neq \alpha \end{array}\right.,$$

where q is set as the level of latent hardening relative to self-hardening, usually between 1 and 1.4. The expression of $h\left(\alpha ,\alpha \right)$ is in a simple form proposed by Peirce, Asaro, and Needleman [45]:

(17) $$h\left(\alpha ,\alpha \right)=h\left(\gamma \right)=h_{0}{\sec h}^{2}\left|\frac{h_{0}\gamma_{\mathrm{tot}}}{\tau_{s}-\tau_{y}}\right|,$$

where $h_{0}$ is the initial hardening modulus, $\tau_{s}$ is the saturated flow stress, and $\gamma_{\mathrm{tot}}$ is the Taylor cumulative shear strain on all slip systems:

(18) $$\gamma_{\mathrm{tot}}=\underset{\alpha }{\sum }\int_{0}^{t}\left|{\dot{\gamma }}^{\alpha }\right|dt$$

By analogy to the relationship between $\sigma_{y}$ and $\tau_{y}$, the $\tau_{s}$ can be calculated approximately with the following formula based on the data in Table 1:

(19) $$\tau_{s}=\tau_{y}+\sigma_{w,\max}/M$$

The uneven height of the local stress and strain of the material is often a sign of failure and destruction, and the accumulated plastic deformation p and local plastic dissipation energy $E_{p}$ are two common physical quantities that predict the location of microcracks in materials [46,47,48]. The expressions of p and $E_{p}$ are as follows:

(20) $$\begin{array}{cc} p=\int_{0}^{T}{\left(\frac{2}{3}l^{p}:l^{p}\right)}^{\frac{1}{2}}dt, & E_{p}=\underset{\alpha }{\sum }\int_{0}^{T}\tau_{\alpha }{\dot{\gamma }}_{\alpha }dt \end{array}$$

In order to determine the plastic behavior of polycrystals, it is necessary to convert the output of the micro-model to the macro-scale, and the macro-scale deformation can be regarded as uniform. The main homogenization models are the Sachs model [49], Taylor model [12], and self-consistent model [50], all of which assume that the stress and strain inside the crystal are uniform.

#### 3.2.2. Polycrystal Morphology and Homogenization Method

Miyamoto’s [51] work shows that coupling the crystal plasticity model to the finite element calculation is a good way to study the confinement effect of adjacent grains. This method takes into account the interaction between grains, satisfying both stress equilibrium and strain compatibility.

In this work, we construct a representative volume element (RVE) and use a homogenization strategy to simulate the macroscopic mechanical behavior of the material, in which the macroscopic quantities are equal to the volume-weighed sum of those over microstructural domains [52]. The macro-scale true stress $\bar{\sigma }$ and strain $\bar{\epsilon }$ are the volume-averaged values computed from the local true stress and strain of the whole domain B, respectively, as follows:

(21) $$\begin{array}{cc} {\bar{\sigma }}_{ij}=\frac{1}{V}\int_{B}\sigma_{ij}dV, & {\bar{\epsilon }}_{ij}=\frac{1}{V}\int_{B}\epsilon_{ij}dV \end{array}$$

### 3.3. Establishment of FE Model

In this work, the commercial finite element software ABAQUS was used to simulate the uniaxial tension of the peak-aged Al-Mg-Si alloy containing β” and β’ precipitates. The constitutive theory of crystal plasticity in Section 3.2 can be realized by using the time integration strategy in the user subroutine UMAT provided by ABAQUS. The UMAT used in this work is mainly based on Huang [53].

**Figure 3:**
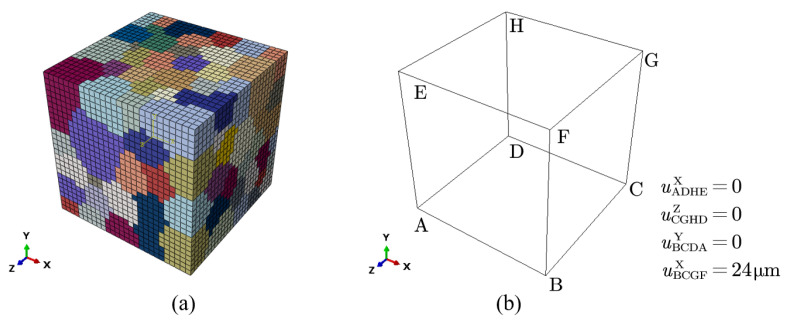
Geometric representation of a finite element model: (**a**) finite element mesh of RVE (19,683 elements, 204 grains); (**b**) boundary condition.

Table 1 shows that the under-aged alloy and the peak-aged alloy have the same grain size, because the grains will not grow when aged at 180 °C. Based on the average grain size obtained from experiments, a square RVE, with a side length of 400 μm, is constructed in Neper [31], which contains a total of 19,683 elements (C3D8 in ABAQUS) and 27 elements in each direction, as shown in Figure 3a. It should be noted that due to the characteristic where Neper can self-define the statistical distribution characteristics of size, the geometric modeling is carried out based on the grain size characteristics obtained through experimental tests, and the average grain size (82.1 μm) and standard deviation (13.6 μm) are consistent with the experimental test values [15]. The orientation of the grains is random, and two sets of different material parameters are set to represent the under-aged alloy and peak-aged alloy. This modeling method is mainly established by Diard et al. [54], which not only takes into account the calculation accuracy, but also ensures that each grain has enough mesh description to reflect the non-uniformity of the internal deformation of the crystal. An engineering strain of 6% (24 μm) is applied to the RVE to simulate uniaxial tensile deformation. Figure 3b shows the boundary conditions of the tensile test. At first, displacements of three adjacent surfaces of the polycrystalline aggregate are fixed. Then, displacement loading is applied on the front surface along the X axis during calculation.

### 3.4. Parameter Calibration

When using the user subroutine UMAT to numerically realize the constitutive relationship established in Section 3.2, there are a total of 9 parameters ($C_{11}$, $C_{12}$, $C_{44}$, ${\dot{\gamma }}_{0}$, m, q, $h_{0}$, $\tau_{y}$, and $\tau_{s}$) that need to be determined, as shown in Table 4. Six of them ($C_{11}$, $C_{12}$, $C_{44}$, ${\dot{\gamma }}_{0}$, m, and q) were from the literature [9,55] and three of them ($h_{0}$, $\tau_{y}$, and $\tau_{s}$) can be assessed according to the experimental stress–strain curve [56,57]. In addition, the values of M and $M_{s}$ in this work are 2.8 and 2, respectively.

**Figure 4:**
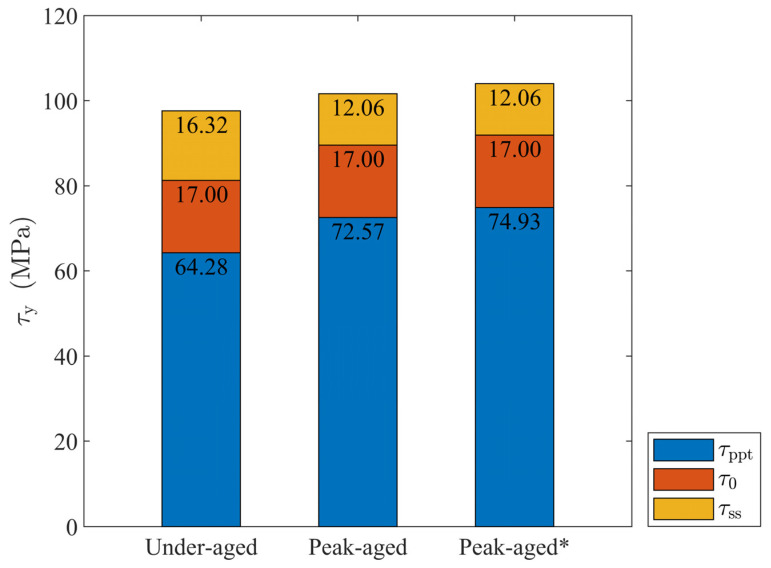
Initial slip resistance of under-aged (β”)/under-aged, peak-aged (β” + β’)/peak-aged, and peak-aged (β”)/peak-aged* alloys.

Considering that the targeted alloy, 6063-T832 alloy, in this work is of the highest strength among the 6063 alloys, it can be approximately assumed that the precipitates are Orowan particles. The precipitation strengthening effect calculation formula proposed by Esmaeili et al. [20] is used to express the contribution of the precipitates as a function of volume fraction, and the superposition of the two precipitation strengthening effects follows the composite criterion proposed by Myhr et al. [39]. The expression for $\tau_{\mathrm{ppt}}$ reads

(22) $$\tau_{\mathrm{ppt}}=\sqrt{{\left(C_{\beta^{\prime }}f_{\beta^{\prime }}{}^{1/2}\right)}^{2}+{\left(C_{\beta \prime \prime }f_{\beta \prime \prime }{}^{1/2}\right)}^{2}},$$

where $C_{\beta^{\prime }}$ and $C_{\beta \prime \prime }$ are the strengthening coefficients of β’ and β” precipitates, respectively, and their values can be obtained as 623.05 MPa and 670.15 MPa based on the volume fraction in Table 1. In the past, whether it was a refined quantitative characterization based on experimental observations, or a simulation and performance prediction of peak-aged precipitates, it was basically used to assume that the peak-aged precipitates had a single composition: only the β” phase. Based on the above parameter calibration results, we consider β’ with a volume fraction of 0.57% in the peak-aged alloy as β” to design the calculation example peak-aged (β”), in order to explore the possible prediction bias caused by the assumption of a single precipitate. Since there is no β’ phase, it can be approximately considered that the two items of $h_{0}$ and $h_{0}/\left(\tau_{s}-\tau_{y}\right)$ are consistent with those of the under-aged alloy, and the key difference is $\tau_{y}$. All parameters are recorded in Figure 4 and Table 4.

**Figure 5:**
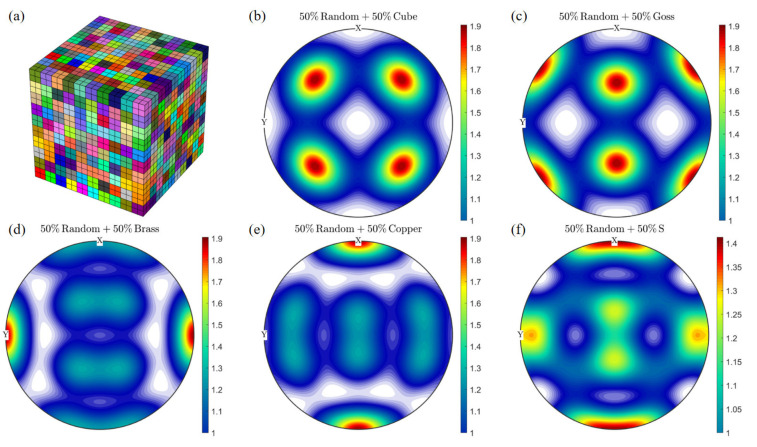
A polycrystalline model with 1000 grains (**a**) and a ${\left\{111\right\}}_{\mathrm{Al}}$ polar diagram of five typical initial orientation features (**b**–**f**).

By comparing the calculation results of peak-aged (β”+β’) and peak-aged (β”) with the experimental results, it is possible to explore the prediction bias caused by the assumption of a single precipitation phase. The overall stress–strain response of RVE is closely related to the orientation characteristics, but is basically unrelated to the morphology of grains and the geometric characteristics of grain boundaries. In order to make the simulation results as inclusive as possible of various realistic orientation characteristics, an RVE containing 1000 grains of the same shape and size was constructed, each represented by eight C3D8 cube elements, as shown in Figure 5. The length of the model will no longer reflect the geometry of the grains. The purpose of this model is to discretize the assumed overall orientation features by using as many grains as possible without too much computational burden, and the geometry of the individual grains is completely consistent to minimize the influence of non-oriented features. The work of Manchiraju et al. [58] has demonstrated the effectiveness of this modeling strategy.

A total of six sets of orientation information are designed. One set is completely random, and five sets of composites of specific orientation and random orientation are designed. This design refers to the work of Choi et al. [59,60] and Mishra et al. [61], in order to take possible anisotropy into consideration as much as possible. The five specific orientations are Cube-$\left(001\right)\left[100\right]$, Goss-$\left(011\right)\left[100\right]$, Brass-$\left(011\right)\left[2\bar{1}1\right]$, Copper-$\left(112\right)\left[\bar{1}\bar{1}1\right]$, and S-$\left(213\right)\left[\bar{3}\bar{6}4\right]$. Figure 5 shows the distribution of five sets of composite orientations on the ${\left\{111\right\}}_{\mathrm{Al}}$ plane. Considering that the maximum stretching amount of the reference alloy 6063-T832 alloy is about 12%, and the crystal plastic finite element method lacks effective mechanism support for the prediction of material failure, we apply boundary conditions with reference to Figure 2, and set the stretching amount as 10% of the side length of the cube, taking the statistical average value of the stress component of RVE in the stretching direction after stretching as the estimated value of the maximum tensile strength, and comparing the results of the two examples of peak-aged(β” + β’) and peak-aged(β”).

## 4. Results and Discussions

**Figure 6:**
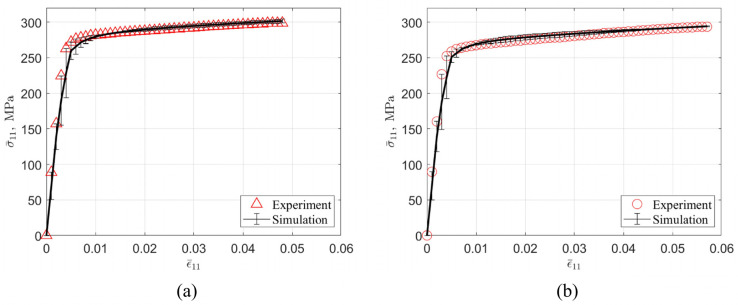
Experimental and simulated uniaxial true stress–strain curves. (**a**) peak-aged alloy; (**b**) under-aged alloy.

Figure 6 shows the true stress–strain curve obtained from the experimental test and the simulation. The error between the simulation results and the experimental values is less than 5%. The difference between the experiment and the simulation is that the grain orientation and morphology of the RVE are different from the actual material, and the state variables used in the crystal plasticity model cannot fully reflect all the material deformation mechanisms. It can be intuitively seen from the figure that the slope of the curve gradually decreases, indicating that the work hardening ability of the material decreases with the increase in deformation. This simulation result is an intuitive response of Formula (17).

**Figure 7:**
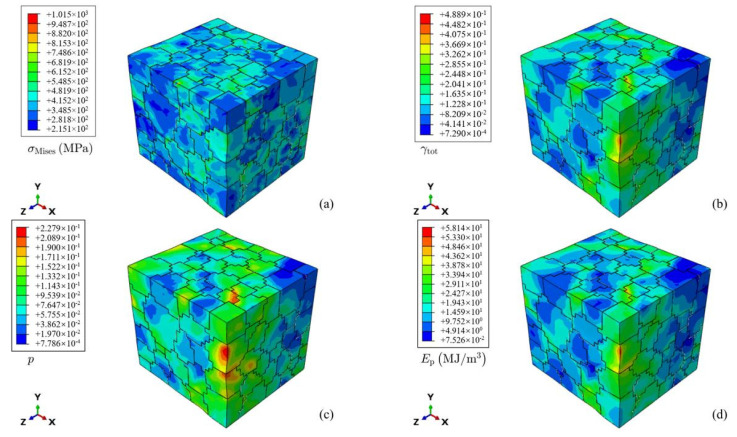
Uniaxial tensile simulation results of peak-aged (β” + β’) alloys: (**a**) Stress component in the tensile direction. (**b**) Strain component in the tensile direction. (**c**) Accumulated plastic deformation. (**d**) Local plastic dissipation energy.

Figure 7 shows the distribution of the Mises stress component $\sigma_{\mathrm{Mises}}$, Taylor cumulative shear strain $\gamma_{\mathrm{tot}}$, cumulative plastic strain p, and plastic dissipation energy $E_{p}$ of the RVE in turn. Figure 7a,b show that the strain deformation and stress response between grains are obviously different, and the stress and strain distribution inside the grains is not uniform. The main reason lies in the elastoplastic anisotropy caused by the difference in grain orientation, so stress and strain concentrations occur when the grain boundary reaches the stress balance and strain coordination at the same time. Combined with the distribution diagram of shear strain, the crystal plastic deformation at the stress concentration can be more intuitively understood. Figure 7c,d show the distribution of p and $E_{p}$, both of which record the history of deformation. In comparison with p, $E_{p}$ more accurately reflects the unevenness of the internal deformation of the grain. Comparing Figure 7b,d, it can be seen that the distribution characteristics of $\gamma_{\mathrm{tot}}$ and $E_{p}$ are consistent with each other.

**Figure 8:**
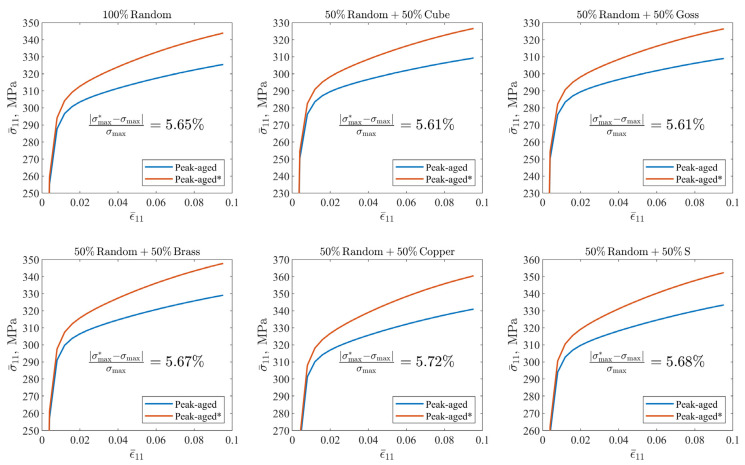
Uniaxial tensile simulation results of peak-aged (β” + β’)/peak-aged and peak-aged (β”)/peak-aged* alloys with different initial orientation characteristics, considering the β” and β’ precipitate composition versus assuming that the precipitate is only β”.

Figure 8 shows the uniaxial tensile simulation results of peak-aged (β” + β’) and peak-aged (β”) alloy. As the deformation increases, the calculated difference between the two materials becomes more and more obvious. The calculation deviation of the maximum tensile strength is around 5.65%, and there is no obvious orientation difference. Even though some of the assumptions made in this work are not absolutely rigorous, the error of the single precipitated phase assumption in the simulation results of crystal plasticity cannot be ignored. The strain hardening behaviors corresponding to different initial textures are also different. Cube and Goss textures are easier to deform than random textures, and Brass textures present an overall mechanical response consistent with random textures. Copper and S textures reflect a stronger deformation resistance than random textures.

## 5. Conclusions

In this work, based on the previous experimental results, a single-crystal strength model considering the grain size, solid solution strengthening effect, and aging strengthening effect is constructed, and the uniaxial stretching process of polycrystalline RVEs is simulated. Two samples with the same composition, the same front-end production process, and only different aging times are selected for comparison to quantitatively distinguish the relative mechanical properties of the two precipitations that play a major strengthening role in peak-aged Al alloys.

The simulated stress–strain curves are in good agreement with the experimental data, confirming that the used constitutive parameters of a single-crystal strength model with two kinds of precipitates are based on real physical implications and thus allow a quantitative description of the deformation behavior at the peak of aging. It is further found that with the increase in aging time, the strength is improved but the deformability is weakened.

By constructing two calculation examples of different precipitated phase compositions and using them to simulate the maximum tensile strength of several materials with typical textures, it is concluded that the two kinds of precipitation should be separately considered when simulating the mechanical response of 6XXX in the future. Compared with past simulation studies that only predict yield strength, the introduction of the crystal plasticity finite element method in the present work can capture richer stress–strain response details and take into account key microstructural information such as real grain geometry and material orientation.

In addition, the findings of this study are not limited to 6XXX aluminum alloys and can help evaluate the production and application of other age-strengthened alloys. The current study presents a preliminary result that has not yet considered material porosity versus microcracks, and different microstructural details will be detailed in the near-future.

## Figures and Tables

**Table 1:** Characterization result of under-aged and peak-aged alloy.

Parameter	Sample-3 h (Under-Aged)	Sample-6 h (Peak-Aged)	Ref.
Matrix compositions (wt.%)	Al-0.25Mg-0.18Si	Al-0.26Mg-0.08Si	[15]
f (%)	$\beta^{\prime \prime }$--0.92	$\beta^{\prime \prime }$--0.68, $\beta^{\prime }$--0.57	[15]
$r_{p}$ (nm)	$r_{\beta^{\prime \prime }}$--3.34	$r_{\beta^{\prime \prime }}$--2.69, $r_{\beta^{\prime }}$--2.84	[15]
d (μm)	82.1	82.1	[15]

**Table 2:** Simulation results of uniaxial tensile test.

Parameter	Sample-3 h (Under-Aged)	Sample-6 h (Peak-Aged)
$E\left(\mathrm{MPa}\right)$	71,902.1	67,898.0
$\sigma_{y}\left(\mathrm{MPa}\right)$	258.6	273.3
$\epsilon_{y}\left(\%\right)$	0.50	0.53
$\sigma_{\max}\left(\mathrm{MPa}\right)$	294.0	299.0
$\epsilon_{\max}\left(\%\right)$	5.74	4.84
$\sigma_{w,\max}\left(\mathrm{MPa}\right)$	35.4	25.7

**Table 4:** Material constants defined in the UMAT subroutine.

Materials	$C_{11},C_{12},C_{44}\left(\mathrm{MPa}\right)$	${\dot{\gamma }}_{0}\left(s^{-1}\right)$	m	q	$h_{0}\left(\mathrm{MPa}\right)$	$\tau_{y}\left(\mathrm{MPa}\right)$	$\tau_{s}\left(\mathrm{MPa}\right)$
under-aged (β”)	106,430, 60,350, 28,210	0.001	0.02	1.4	40.0	97.60	110.24
peak-aged (β” + β’)	106,430, 60,350, 28,210	0.001	0.02	1.4	40.0	101.63	110.81
peak-aged (β”)	106,430, 60,350, 28,210	0.001	0.02	1.4	40.0	103.99	116.63

## Data Availability

The data presented in this study are available on request from the corresponding author.
